# Changes in Composition of the Gut Bacterial Microbiome after Fecal Microbiota Transplantation for Recurrent *Clostridium difficile* Infection in a Pediatric Heart Transplant Patient

**DOI:** 10.3389/fcvm.2017.00017

**Published:** 2017-04-04

**Authors:** Kyle L. Flannigan, Taylor Rajbar, Andrew Moffat, Leanna S. McKenzie, Frank Dicke, Kevin Rioux, Matthew L. Workentine, Thomas J. Louie, Simon A. Hirota, Steven C. Greenway

**Affiliations:** ^1^Department of Physiology and Pharmacology, Cumming School of Medicine, University of Calgary, Calgary, AB, Canada; ^2^Department of Immunology, Microbiology and Infectious Diseases, Cumming School of Medicine, University of Calgary, Calgary, AB, Canada; ^3^Snyder Institute for Chronic Diseases, University of Calgary, Calgary, AB, Canada; ^4^Department of Paediatrics, Cumming School of Medicine, University of Calgary, Calgary, AB, Canada; ^5^Alberta Children’s Hospital Research Institute, University of Calgary, Calgary, AB, Canada; ^6^Libin Cardiovascular Institute of Alberta, University of Calgary, Calgary, AB, Canada; ^7^Department of Medicine, University of Calgary, Calgary, AB, Canada; ^8^Faculty of Veterinary Medicine, University of Calgary, Calgary, AB, Canada; ^9^Department of Biochemistry and Molecular Biology, University of Calgary, Calgary, AB, Canada

**Keywords:** microbiome, heart transplantation, immunosuppression, pediatric, fecal microbiota transplant

## Abstract

The microbiome is increasingly recognized as an important influence on human health and many of the comorbidities that affect patients after solid organ transplantation (SOT) have been shown to involve changes in gut bacterial populations. Thus, microbiome changes in an individual patient may have important health implications after SOT but this area remains understudied. We describe changes in the composition of the fecal microbiome from a pediatric heart transplant recipient before and >2.5 years after he underwent repeated fecal microbiota transplantation (FMT) for recurrent *Clostridium difficile* infection (CDI). With both documented episodes of CDI, there was marked loss of bacterial diversity with overgrowth of Proteobacteria (>98.9% of phyla identified) associated with symptomatic colitis that was corrected after FMT. We hypothesize that a second CDI occurring after FMT was related to incomplete restoration of normal bowel flora post-FMT with relative deficiencies of the phyla Firmicutes and Bacteroidetes and the families *Lachnospiraceae* and *Ruminococcaceae*. Following the second FMT, there was a gradual shift in gut bacterial composition coincident with the recipient developing lymphonodular hyperplasia of the colon and painless hematochezia that resolved with discontinuation of mycophenolate mofetil (MMF). This case documents dynamic changes in the bacterial microbiome after FMT and suggests that MMF may influence the gut microbiome with consequences for the patient.

## Introduction

Changes in the microbiome, the collection of microbes co-existing with the human host, may influence patient outcome after solid organ transplantation (SOT) (1, 2) but examples are lacking, particularly in children. We (unpublished data) and others have noted significant changes in the murine gut microbiome in response to mycophenolate mofetil (MMF) and other immunosuppressive drugs (3, 4). Increased susceptibility to infection is a known complication following SOT and the rate of *Clostridium difficile* infection (CDI) in SOT patients may be up to five times higher in comparison to other hospitalized patients (5). Antibiotics are a commonly reported risk factor for CDI due to depletion of protective bacteria, and reconstitution with fecal microbiota transplantation (FMT) is a promising therapeutic option that has been successfully applied to SOT patients and children (6, 7). Immunosuppression causing changes in the gut microbiome could potentially contribute to increased risk for CDI, influence the host response to CDI and FMT or increase the risk for developing complications post-SOT.

Utilizing 16S rRNA sequencing of serial stool samples, we followed the course of a pediatric heart transplant recipient who developed recurrent CDI treated with FMT on two separate occasions, by enema and oral capsules. Subsequent development of colonic lymphonodular hyperplasia was attributed to MMF. We describe changes in the composition of this patient’s fecal microbiome over time and relate them to clinical symptoms.

## Case Report

A 5-year-old male presented with a 1-year history of diarrhea that had progressed over the previous month to become bloody. The patient had undergone ABO-incompatible (donor AB, recipient A) heart transplantation at 10 months of age for idiopathic dilated cardiomyopathy and had been maintained on tacrolimus (levels of 5–7 μg/L at the time of presentation) and MMF (20–30 mg/kg/day divided BID). Stool was positive for *C. difficile* toxin, and he was treated with oral metronidazole. Transient improvement was noted but symptoms (bloody and watery diarrhea, abdominal cramping, increased flatulence) recurred once the antibiotics were stopped and stool again tested positive for *C. difficile* antigen and the toxin B gene. He was treated with oral vancomycin and had normal stools but symptoms recurred with cessation of antibiotics. He subsequently experienced three relapses despite treatment with vancomycin, nitazoxanide, and Florastor^®^ (probiotic containing *Saccharomyces boulardii*) with symptoms returning 4–14 days after stopping antibiotics. Due to the recurrent CDI, the patient underwent FMT *via* enema with stool donated from his healthy asymptomatic mother (8). The procedure was well tolerated with no complications and the patient’s stools normalized within 1 week.

He remained clinically well until presenting 3 months later with fever and painful swelling of the left neck. He was diagnosed with cervical adenitis and treated with intravenous cefotaxime and clindamycin. After 1 week, the patient was discharged home on amoxicillin and clavulanic acid to complete a total of 14 days of antibiotic therapy. He then presented 3 months later with a 3-week history of increasing amounts of blood in the stool, abdominal cramping, increased flatulence, foul smelling, and slightly loose stools (not large watery stools as previously pre-FMT). Stool was positive for *C. difficile* toxin by PCR testing for the toxin B gene, and he was treated with oral vancomycin. The diarrhea persisted and he underwent a second FMT, from the same donor but this time using oral capsules (unpublished data), 253 days after his initial presentation with resolution of symptoms.

After the second FMT, the patient remained clinically well with normal stools but presented to the Gastroenterology clinic 2 years after his first presentation with streaks of bright red blood passed per rectum. He had no diarrhea or abdominal cramping and his stools were described as normal and were negative for *C. difficile* toxin. He was noted to have perianal erythema and underwent sigmoidoscopy followed by colonoscopy. Endoscopy revealed non-specific lymphonodular hyperplasia and very mild eosinophilic colitis. His painless hematochezia persisted despite treatment with prednisone, ketotifen, intrarectal 5-ASA, and hydrocortisone foam but slowly resolved after the MMF was stopped and he was switched to sirolimus. Six months after stopping the MMF, all symptoms had resolved and he has remained clinically well with no recurrence of GI symptoms on a regimen of tacrolimus (target levels of 5–7 μg/L) and sirolimus (target levels of 3–5 μg/L).

## Materials and Methods

### Clinical Specimens

This study was approved by the Conjoint Health Research Ethics Board at the University of Calgary (study ID REB13-0576). All subjects gave written informed consent in accordance with the Declaration of Helsinki. Stool samples were collected from the donor and patient/recipient over a 2.6-year period (days 0–967 after his initial presentation) and stored at −80°C until processed. DNA was extracted from feces using mechanical and enzymatic digestion followed by phenol:chloroform extraction and cleanup as previously described (9). Purified DNA was used for PCR amplification of the bacterial 16S rRNA gene V3/V4 regions (10). Libraries were constructed and sequenced on an Illumina MiSeq in the Nicole Perkins Microbial Communities Core Laboratory at the University of Calgary.

### Data Analysis

Processing was done using the UPARSE pipeline (11). Taxonomy was assigned to the representative sequences using the RDP naive Bayesian classifier (12). Downstream analysis of the final OTU table was done in R using phyloseq 1.16.2 (13). Beta diversity was evaluated using the weighted UniFrac distance metric (14) on normalized OTU counts and visualized with non-metric multidimensional scaling. OTUs that differed significantly between the donor and the two recipient groups were identified using generalized linear models (15).

## Results

**Figure 1:**
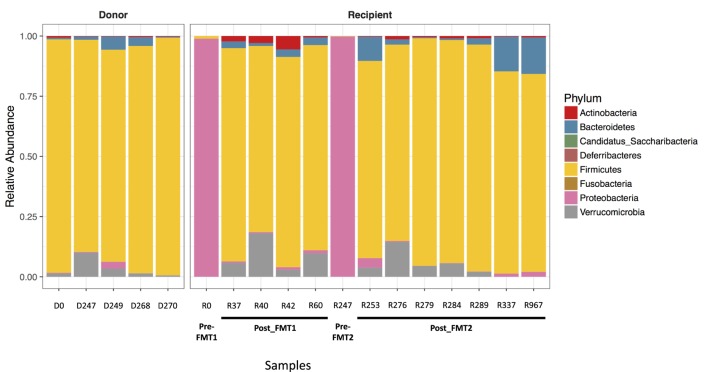
**Proteobacteria predominates with *Clostridium difficile* infection (CDI)**. Bar plots showing the relative abundance of bacterial phyla in stool samples collected from the donor and recipient before and after fecal microbiota transplantation (FMT). The five donor samples are stable with a predominance of Firmicutes (yellow). When the patient has symptomatic CDI, there is an almost complete replacement of normal stool flora (dominated by Firmicutes) with Proteobacteria (pink).

Examination of the different bacterial phyla in the patient’s feces when he was symptomatic with CDI, revealed that Proteobacteria almost completely dominated (>98.9% of identified phyla) and had replaced the normal microbiome, which consists primarily of members of the phylum Firmicutes as seen consistently in the five donor samples and in the patient’s post-FMT samples (Figure 1). With symptomatic CDI, the predominant genera were *Klebsiella* and *Escherichia/Shigella* (data not shown). After FMT, the patient’s microbiota was no longer dominated by Proteobacteria and assumed a composition resembling that seen in the donor samples. These changes coincided with the resolution of his CDI-related symptoms.

**Figure 2:**
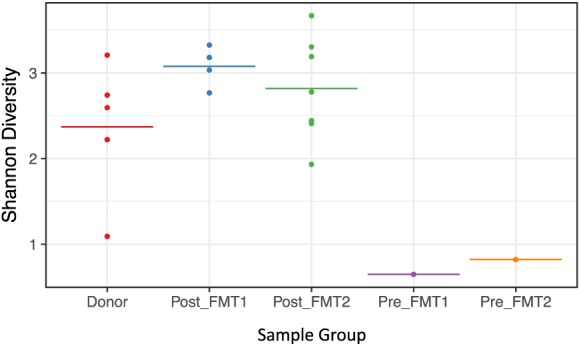
**Loss of alpha diversity with *Clostridium difficile* infection**. Shannon (alpha) diversity reflects diversity within a sample. For the five sample groups examined (Donor, Post_FMT1, Post_FMT2, Pre_FMT1, and Pre_FMT2), there is a marked loss of bacterial diversity within the two samples prior to fecal microbiota transplantation (FMT) compared to the pooled Donor and Post-FMT samples that show comparable diversity.

**Figure 3:**
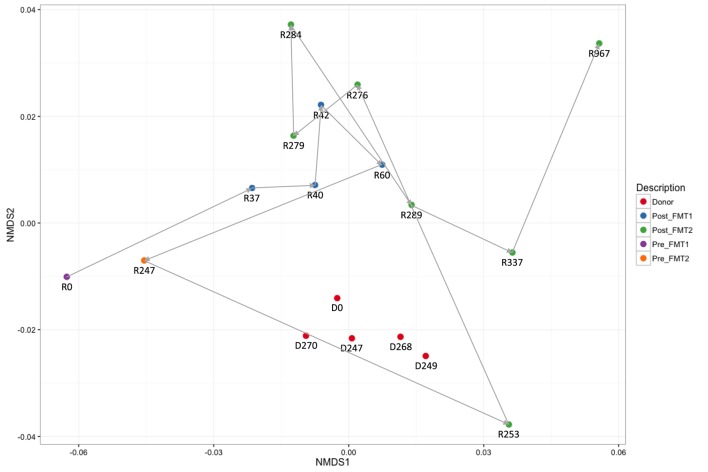
**Dynamic beta diversity in our pediatric transplant patient**. Changes in beta diversity reflect changes in composition between samples. The non-metric multidimensional scaling (NMDS) plot shows changes in beta diversity for donor and recipient stool samples over time. The five donor samples (red circles) remain clustered together with stable diversity but there are large changes in the patient samples before (purple and yellow circles) and after fecal microbiota transplantation (FMT) (blue and green circles).

Active CDI resulted in a marked decrease in alpha diversity that was reversed by FMT (Figure 2). Looking at changes in beta diversity over time (Figure 3), we see that both samples collected from the patient at the time of CDI (R0, Pre-FMT1 and R247, Pre-FMT2) cluster closely together. After the first FMT, the stool composition remained relatively tightly clustered (blue circles) but was distinct from that of the donor. The second FMT initially caused the stool composition to shift towards the donor (R253) but we see that, over a relatively short period (23 days until R276), there is a shift away from the donor and back toward the post-FMT1 samples. However, during the 2 years after the second FMT, the patient samples became increasingly distinct from the donor and the early post-FMT samples. This shift coincides with the patient developing painless hematochezia and apparent MMF-related lymphonodular hyperplasia of the colon.

**Figure 4:**
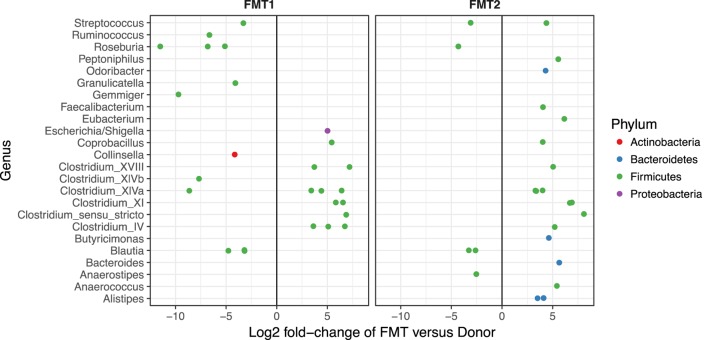
**Deficiencies in bacterial families after the first fecal microbiota transplantation (FMT)**. Differential abundance (*x*-axis) of bacterial families (*y*-axis) in patient/recipient stool relative to the donor after the first (FMT1) and second FMT (FMT2). Within the phylum Firmicutes, there are relative deficiencies of the families *Ruminococcaceae* and *Lachnospiraceae* (indicated by increased number of green circles to the left of 0) and the phylum Bacteroidetes (blue circles) after FMT1.

Although after each FMT alpha diversity is restored and the patient samples cluster together with respect to their beta diversity (blue and green circles in Figure 3), there were distinct differences in the relative abundances of the bacterial phyla post-FMT1 and post-FMT2 (Figure 4). After the first FMT, the patient remained deficient in bacteria from the phylum Bacteroidetes and relatively deficient in Firmicutes with a particular paucity of the bacterial families *Ruminococcaceae* and *Lachnospiraceae*.

## Discussion

In our patient, a pediatric heart transplant recipient, we have described the dynamic shifts in the intestinal microbiota that occurred with CDI, therapeutic FMT, and chronic MMF exposure. Others have noted that CDI is associated with disruption of the gut microbiome, specifically a loss of overall diversity and alteration in microbial composition with a dominance of Proteobacteria (16), which is consistent with our observations. Given that *C. difficile* is a member of Firmicutes, the Proteobacteria do not represent CDI but rather the associated dysbiosis. The relative deficiency of beneficial members of Firmicutes (e.g., butyrate-producing *Lachnospiraceae* and *Ruminococcaceae*) has been seen after failed FMT in patients with CDI and in asymptomatic *C. difficile* carriers (16, 17) and may have contributed to the fragility of the microbiome after FMT1 for our patient. The need for repeat FMT may be more common in the SOT population. Both patients reported by Friedman-Moraco et al. required repeat FMT for complete resolution of their diarrhea symptoms and the success rate for FMT in immunocompromised patients was only 79% compared to an initial success rate of 91% in immunocompetent individuals (18, 19). It is speculated that SOT recipients may have more severe dysbiosis with damaged microbiota due to immunosuppression and antibiotic use and sequential FMT may be necessary to correct the alterations in the intestinal microbiota (18).

Fecal microbiota transplantation acts to restore Bacteroidetes and Firmicutes and eliminates Proteobacteria (16), consistent with our findings. Looking at bacterial abundance more closely, we see that before the first FMT, there was a predominance of the Genus *Klebsiella*. However, when his symptoms recurred and just before receiving the second FMT, the dominant genera included both *Klebsiella* and *Escherichia/Shigella*. This difference may be related to his treatment with antibiotics but could also be related to the incomplete restoration provided by the first FMT.

The donor microbiota remained relatively stable over the 9 months of the available samples. This stability contrasts with the dynamic changes of the patient samples over time. The patient’s samples cluster both pre- and early post-FMT in distinct groups from the donor with only a single sample 4 days post-FMT2 (R253) being closely associated with the donor cluster. By 3 months post-FMT2, the patient samples (R337 and R967) have become distinct from both the donor and the early post-FMT samples. We ascribe at least some of these changes to the patient’s immunosuppression, with MMF being the most likely candidate.

Eight months after successful treatment of recurrent CDI with the second FMT, the patient developed painless hematochezia related to lymphonodular hyperplasia of the colon. Although the patient had a strong history of atopy, he did not respond to multiple anti-inflammatory therapies and ultimately only improved after the MMF was discontinued. We suggest that in this case the MMF was responsible for both the colonic tissue changes, which are non-specific and have been associated with infection and allergy in children (20), and changes in the microbiome. However, this conclusion remains speculative since this is only an observational study and population composition at the phylum level as shown in Figure 1 is clearly inadequate to illustrate this change. Studies are currently ongoing in our laboratory to explore the relationship between immunosuppressive drugs and the microbiome.

## Conclusion

We have presented sequential changes in the gut microbiome of a pediatric patient who developed recurrent CDI after heart transplantation and was successfully treated with two separate FMTs 249 days apart. Changes in bacterial composition are consistent with prior observations in immunocompetent individuals, but we hypothesize that at least some of the changes observed are related to the effects of his chronic immunosuppression, particularly MMF, on the gut microbiome. This single observation remains to be confirmed in larger patient series.

## Author Contributions

SH and SG designed the study. LM, FD, and TL managed the patient and collected the samples. TR, AM, and KR isolated DNA and prepared the samples for sequencing. MW performed the data analysis. KF and SG wrote the manuscript. All authors reviewed the manuscript.

## Conflict of Interest Statement

The authors declare that the research was conducted in the absence of any commercial or financial relationships that could be construed as a potential conflict of interest.

## Abbreviations

CDI, *Clostridium difficile* infection; FMT, fecal microbiota transplantation; MMF, mycophenolate mofetil; SOT, solid organ transplantation.
